# Negotiating the impact of international experiences on professional identity development: A case study of Chinese college English teachers

**DOI:** 10.3389/fpsyg.2022.1007649

**Published:** 2022-10-05

**Authors:** Fengjuan Zhang, Jing Wang

**Affiliations:** School of Foreign Language Education, Jilin University, Changchun, China

**Keywords:** professional identity, study abroad, EFL teachers, returnee teachers, China

## Abstract

This study traced changes in Chinese college English teachers’ professional identities as a result of participating in international professional development programs and examined how the teachers negotiated their professional identities upon return to China. Five college English teachers with at least 10 months of overseas professional development experience took part in the study. Data were collected through semi-structured interviews and relevant documents. It was found that international experiences had a great impact on the teachers’ professional identity construction by empowering them to develop multiple identities as language teaching professionals, university academics, and change agents. The teachers’ reconstruction of professional identities upon return to China was not a linear and smooth process. It was a dynamic process of negotiating with the constraints of the personal and professional contexts to which the teachers returned. The study sheds light on the identity development of internationally trained language teachers and contributes to a deeper understanding of the impact of international experiences on the professional development of second language teachers in similar contexts. It has implications for study abroad programs and for home institutions about how to support the long-term professional development of returnee teachers.

## Introduction

In the field of second language teaching, it is a common practice for institutions in many countries to send their language teachers for short-term or long-term study abroad programs. Research has shown that study abroad experiences can bring positive changes to language teachers’ knowledge, beliefs, and practice (Allen, 2010; Trent, 2011; Li and Edwards, 2013; Mayumi and Hüttner, 2020). These changes may contribute to the construction of new professional identities for teachers, which will eventually affect students’ learning experiences and learning outcomes. Despite many reports on the linguistic, cultural, and pedagogical benefits of international programs for language teachers, very limited research has examined the impact of international programs on the professional identity development of the participating teachers, especially the identity reconstruction process of negotiating new learning with the local context after the teachers return to their home country. Such exploration can be of significance in achieving insights into how study abroad experiences are translated into teachers’ professional growth in their specific work context.

This study aims to explore the impact of international experiences on the professional identity development of Chinese college English teachers by tracing the teachers’ identity changes as a result of participating in international professional development programs. Particular attention is also devoted to examining how the teachers reconstruct their professional identities upon return to China as they negotiate between their newly acquired global perspective and the local constraints. The identity tensions and conflicts that the teachers may experience upon their re-entry into the workplace, and the creative strategies that they adopt to resolve tensions and conflicts, are closely examined. The study may shed light on the identity construction of internationally trained language teachers and contribute to a deeper understanding of the impact of international experiences on the professional development of second language teachers in similar contexts.

## Literature review

Study abroad (SA) is an important topic in the field of second language teaching and learning (Freed, 1995, 1998; Kinginger, 2009; Isabelli-García et al., 2018; Kang and Pacheco, 2021). The majority of this body of research has centered on students’ perspectives (Freed, 1995; Taguchi and Collentine, 2018). Over the past two decades, as more and more second language teachers participate in international professional development programs, increasing research attention has been devoted to the experiences of language teachers in study abroad contexts (Trent, 2011; Çiftçi and Karaman, 2019; Baecher, 2020). Research has shown that language teachers studying abroad constitute pre-service and in-service teachers across various educational levels in different settings. Pre-service language teachers with limited or no teaching experience usually take part in study abroad programs as part of their second language teacher education to acquire professional knowledge and prepare for future teaching. Their study abroad experiences have been extensively studied in the field (Çiftçi and Karaman, 2019). On the other hand, in-service teachers in study abroad contexts have received far less research attention (Allen, 2010; Baecher and Chung, 2020). With prior teaching experience and established beliefs about teaching and learning, in-service teachers may attend study abroad programs for diverse purposes and may have different feelings, understandings, experiences, and outcomes when compared with pre-service teachers, which deserves further investigation.

A central concern of study abroad research with language teachers is to investigate the impact of overseas programs on language teachers’ professional development. Studies suggest that well-designed study abroad programs can influence language teachers’ professional development in multiple ways. First of all, study abroad programs can enhance language teachers’ linguistic knowledge, awareness, and competence. In Allen (2010), the 30 teachers of French reported improved language skills and enhanced confidence in speaking the target language after attending a three-week summer institute that took place in France. Wang (2014) surveyed 91 Chinese secondary school English teachers with study abroad experiences and found that most of the teachers rated study abroad as the most useful activity in helping them improve language proficiency, and that the length of stay in English-speaking countries is an important factor in the teachers’ self-assessment of language proficiency. In Choe (2016), the 42 Korean teachers of English reported significant improvement in listening, speaking, and writing after attending a four-week teacher training program in the US, which lends further support to previous studies about the positive outcomes of study abroad programs for teachers’ language development. Because language and identity are interrelated, and the process of identity construction is discursive through language, language competence can exert a crucial influence in shaping language teachers’ self-efficacy and professional capacity. Improvement in language knowledge and skills as a result of international experiences can change language teachers’ conceptions of who they are and how they behave in specific language teaching contexts, thus affecting their professional identity development.

Apart from the linguistic impact, several studies have also revealed the influence of study abroad on language teachers’ cultural knowledge and intercultural competence (Jackson, 2008; Allen, 2010; Larzén-Östermark, 2011). Study abroad experiences are cross-cultural sojourns, and language teachers taking part in study abroad programs are all cross-cultural communicators. International experiences can help language teachers increase cultural knowledge and intercultural sensitivity, promote cultural understandings, and construct cross-cultural identity. For example, the pre-service and in-service teachers from the United States in Nero’s (2009) study developed more thorough understandings of the language learner, the language learning process, and the target culture in a four-week overseas seminar held in the Dominican Republic, which contributed to the teachers’ development of culturally responsive pedagogy. The teachers of French in Allen (2010) expanded their knowledge of cultural products, practices, and perspectives after their study abroad experience in France. In Suzuki (2021), the cohort of Japanese pre-service teachers of English who undertook study abroad teacher training in the US developed more flexible views of English for intercultural communication. In these studies, the teachers’ cultural gains during study abroad played an important role in their teacher education and development.

Study abroad can also bring about pedagogical benefits by changing language teachers’ cognition and teaching practice. Relevant studies have shown that study abroad may change teachers’ beliefs about teaching goals, teaching methods, instructional materials, and so forth, while fostering new understandings of students and teachers themselves (Nero, 2009; Wang, 2014; Mayumi and Hüttner, 2020). These changes in teacher cognition may in turn cause changes in teaching practice. For example, Allen (2010) found that the teachers of French used the authentic materials they brought back and the knowledge they had acquired during study abroad in France to improve their instruction, even though they did not make dramatic changes in their curricula. Li and Edwards (2013) found that Chinese EFL teachers selectively implemented new teaching approaches and methods they had learned from a professional development program in the United Kingdom. The returnee teachers changed their roles from authority figure to supporter, guide, and motivator, turning student centered rather than teacher centered in curriculum design and classroom planning. In a similar vein, Macalister (2016) examined the impact of 2 years of transnational study in New Zealand on the language teaching practice of two Malaysian pre-service teachers, identifying some apparent influences such as signs of a student-centered approach to teaching and classroom management techniques. These studies suggest that overseas training could positively impact participating teachers’ pedagogical beliefs and instructional practices.

The above review of literature suggests that study abroad experiences have the potential to enhance language teachers’ knowledge base and professional competence, which may in turn affect teachers’ professional identity development. Identity, which can be defined as “our understanding of who we are and who we think other people are” (Danielewicz, 2001, p.10), is dynamic, complex, multifaceted, and socially constructed (Norton, 2013). It is constantly changing with the current demands. In the study abroad environment, teachers’ identities are likely to change and reconstruct to meet the needs at the moment. The high frequency of using the target language, the anxiety and risks in exposure to and adapting to new ways of living, teaching, learning, and thinking, and the potential conflicts and negotiations between different cultural traditions and realities, all may impact the teachers’ sense of self and perceptions as language teachers, leading to the construction and reconstruction of teacher identities in study abroad contexts. What’s more, upon return to home country, the returnee teachers may continue to encounter identity issues and experience tensions as they reconcile the newly acquired professional identities with the realities of the teaching context. In a nutshell, identity is central in understanding the impact of study abroad on language teachers’ professional development.

So far, researchers have examined the construction of language teacher identity in diverse contexts, such as the classroom contexts (Kanno and Stuart, 2011; Hiver and Whitehead, 2018), the broader institutional and social contexts (Tsui, 2007; Wang and He, 2022), the online contexts (Stranger-Johannessen and Norton, 2017; Chao, 2022), the educational innovation contexts (Tao and Gao, 2018; Kessler, 2021; Dikilitaş and Bahrami, 2022), and various formal and informal learning contexts (Trent, 2013; Yuan and Mak, 2018; Jeongyeon and Hye Young, 2020; Banegas et al., 2022). However, there has been a paucity of research attention on the construction of language teacher identity in study abroad contexts, although study abroad has generally been considered an important tool for teacher learning with positive outcomes for the professional development of language teachers, as the above review of relevant works has shown. Among the few studies examining language teacher identity construction during study abroad, Trent (2011) found that the eight Hong Kong pre-service teachers attending an Australian training program managed to reconcile the trajectories of teacher identity in the past, present, and future. Kasun and Saavedra (2016) studied eight pre-service teachers from the US who participated in a four-week residency program in Mexico, finding that the teacher candidates experienced three salient identity shifts: being “socially aware,” being “empaths,” and being “creator of a loving learning space” rather than “classroom managers” (pp. 13–14). Although very limited in number, this line of research on language teachers’ transnational, intercultural experiences and identity development resulting from participation in study abroad programs shows that international experiences can exert a powerful influence on language teachers’ professional identity development. This influence is ongoing, since identity issues and tensions may continue to arise as the teachers complete their study abroad program and return to home country. The complicated long-term impact of study abroad on language teacher identity development needs to be better understood.

This study set out to explore the impact of international programs on the professional identity development of Chinese college English teachers and to examine the process of identity negotiation upon the teachers’ return to home country. It was guided by the following research questions:

1. What changes in professional identities did the Chinese college English teachers go through as a result of their international experiences?
2. How did the Chinese college English teachers negotiate their professional identities upon return to China?

## Materials and methods

### Research design

This study employed a qualitative case study design (Yin, 2009, 2018). Case study can help “understand the complexity and dynamic nature of the particular entity,” and “discover systematic connections among experiences, behaviors, and relevant features of the context” (Johnson, 1992, p. 84). It is best suited to answer “how” and “why” questions in real-life events within specific contexts (Yin, 2009). By adopting a case study design, this study aimed to obtain a thorough understanding of the impact of international experiences on the professional identity development of Chinese college English teachers and to achieve insights into the complex and dynamic process of professional identity reconstruction upon the teachers’ return to China.

### Participants

The participants were five Chinese college English teachers with overseas professional development experience. Variation was considered when recruiting the participants by convenience sampling so that they represented different educational backgrounds and different international experiences. The international programs that the teachers participated in included Singapore Nanyang Technological University (NTU) English Teachers’ Training Program and a series of teacher study abroad programs funded by China Scholarship Council (CSC). The length of time spent in the overseas programs was from 10 months to 6 years. Most of the participants were female, with only one male, reflecting the predominance of women in the college English teaching force in China. Table 1 presents the background information of the participants.

**Table 1:** Background information of the participants.

Name	Gender	Years of teaching	Educational background	Overseas experience
Jessica	F	10	MA in applied linguistics	Visiting scholar, United States, 2 years.
Sue	F	17	PhD in Chinese literature	CSC Study Abroad Program for Young Backbone Teachers, United States, 1 year.
Lucy	F	15	PhD in linguistics	CSC Overseas Doctoral Program for University Teachers, United Kingdom, 6 years.
Jane	F	15	MA in applied linguistics	CSC Study Abroad Program for Young Backbone Teachers, The Netherlands, 1 year.
Tony	M	9	MA in English literature	Singapore NTU English Teachers’ Training Program, 10 months.

### Data collection

Data sources for this study included semi-structured interviews with the five teachers and collection of relevant documents such as teachers’ reflective journals and study abroad reports. The main data source is semi-structured interviews, which can provide rich, thick descriptions of the participants’ real-life experiences and insights into the complexities and intricacies of the issues under investigation (Wengraf, 2001; Seidman, 2006). The interview with each participant lasted 1 to 2 h, in which the participant talked about their international professional development experiences, their attitudes and feelings toward their international experiences, the gains and changes that international experiences brought to them, the challenges they encountered after returning to China, and the strategies they adopted to deal with tensions and challenges as they returned to their positions.

### Data analysis

The qualitative data were analyzed using thematic analysis (Braun and Clarke, 2006). First, the transcribed interviews and relevant documents were read several times to obtain a general understanding of each participant’s experiences. Two major categories were produced in reference to the research questions, which are identity change and identity negotiation. Then the data for each participant were coded into these two broad categories and refined to create sub-categories and specific codes. Next, cross-case analysis was undertaken to examine the similarities and differences between the five cases and to identify salient themes and recurrent patterns across the cases (Miles and Huberman, 1994; Merriam, 2009). For instance, under the category of identity change, we generated codes such as craftsman, professional, researcher, academic, and change agent. After several rounds of close reading of the coded data within each case and constant comparison between the cases, we identified three salient themes in teachers’ identity change, which are from craftsman to language teaching professional, from teacher to university academic, and from follower to change agent. This way, the data from the five participants were coded, compared, cross-examined, and integrated for report based on the central concerns of this study and relevant literature.

## Findings and discussion

Analysis of data showed that international experiences had a significant impact on Chinese college English teacher’s professional identity development. It was “an eye-opener” (Jessica) and enabled the teachers to “turn over a new leaf in life” (Sue). It helped the teachers re-orient their profession and allowed them to develop multi-faceted identities as empowered language teaching professionals, academics with clear directions of research, and agents of change. However, the process of professional identity reconstruction for the returnee teachers was not a linear and smooth process. It was full of tensions, struggles, adjustments, and compromises. In the following, the impact of international experiences on the returnee teachers’ professional identity development and the teachers’ negotiation of professional identity tensions upon return to their positions are presented and discussed.

## Changes in teachers’ professional identities

### From “craftsman” to language teaching professional

Before going abroad, several of the teachers had vague ideas about what it means to be a language teaching professional. International experiences provided them with multiple opportunities and rich resources to acquire new knowledge and skills and re-examine their cognition about various aspects of teaching. Specifically, international professional development experiences not only improved their English language proficiency and intercultural competence, but also broadened their teaching repertoires and clarified their teaching conceptions, all of which enhanced their knowledge base, changed their understanding of language teaching, and empowered them to pursue teaching excellence. Teaching was transformed from the mere job of a “craftsman” to a career of a language teaching professional. According to some teachers, the identity transformation from mere teachers to empowered language teaching professionals was a very important gain in their international experiences.

Lucy described how living and studying in Britain for several years greatly enhanced her professional competence:

From the perspective of teaching, the change is needless to say. As an English teacher, I lived in an English-speaking country, a pure English environment for so many years, to learn, to feel the influence of the surrounding language and culture, and to mingle into the local culture. My language awareness, language ability, cultural awareness and understanding have been totally transformed. Before I went abroad, the teaching materials I used with the students were like second-hand information. I passed on the information to the students just like a broker. But now, I have obtained first-hand information through my experience of living abroad. The effect of instruction is very different from that of the past.

Although the teachers’ report of gains in cultural knowledge varied depending on the country they visited, all the teachers agreed that being immersed in an English-speaking environment improved their English proficiency and increased their confidence in using English. As non-native speakers of English and learners of English themselves, EFL teachers such as the teachers in this study tend to attach great importance to language proficiency, which concerns whether they have credibility as qualified English teachers. In this sense, linguistic gains from study abroad can promote teachers’ self-efficacy and professional capacity, which in turn affects the development of teachers’ professional identities.

Jessica’s professional life was also transformed by her overseas experience. Two years of being a visiting scholar in the US, during which time she availed herself of every opportunity to learn, turned her into a passionate, confident, and reflective language teaching professional. She believed that international experiences are highly necessary for the professional development of language teachers, and that the returnee teachers should not view their work as simply a means of making a living, which, she noted, would be a waste of international experience. Her teaching journals, which she kept regularly after each class for quite some time after her return to China, were full of reflections on how to improve teaching and proactively respond to the college English reforms in China that were affecting the work prospects of thousands of college English teachers. When she talked about the impact of international experiences for herself, she confirmed the linguistic and cultural gains and also cited several pedagogical changes, especially changes in the understanding of her role from a knowledge transmitter to a guide and facilitator for student learning. With this new understanding of her role in relation to the students, her teaching approach saw a shift from teacher centered to student centered. Specifically, her English class used to consist of typical traditional teaching activities and routines: she would engage in paragraph-to-paragraph explanation of grammar and language points in the text and her students were supposed to read aloud the text, listen to the teacher’s instruction, and do relevant exercises. After she returned from the US and resumed teaching, her students texted that she had created a “totally new and different English learning environment” for them, which they enjoyed immensely. For example, according to her teaching journals, she would pose several questions for the students to consider and discuss before dealing with the text. Her way of dealing with the text also changed from teacher explanation to students’ collaborative learning among groups before she answered the students’ questions and engaged the students in higher-order learning activities.

These findings confirm previous studies about the positive impact of study abroad on language teachers in promoting teachers’ language proficiency, cultural knowledge, and pedagogical skills (Allen, 2010; Trent, 2011; Li and Edwards, 2013; Mayumi and Hüttner, 2020). These areas are the core components of language teachers’ knowledge base. Improvements in these areas can enhance language teachers’ expertise and self-image, contributing to teachers’ development of professional identities.

#### From teacher to university academic

In this study, overseas experiences provided the teachers with valuable opportunities to foster research awareness, to get trained in theory and research methodology in their field of study, and to network with international scholars, all of which helped them embark on an academic career as both a teacher and a researcher in the university context. For many of the teacher participants, this newly acquired academic identity was the biggest gain from their overseas experience. As teaching constituted a large proportion of their daily work and was their main concern at work, several teacher participants developed a research interest in language teaching and learning, becoming teacher researchers.

Jane used to have no awareness of research. Although many of her colleagues engaged in research activities and paper publishing in order to achieve career advancement, she could not find motivation to do research for this purpose. During her stay in the Netherlands, however, Jane started to see the intrinsic connections between teaching and research. By observing the practice of international colleagues around her, she realized the value of teacher research for informing and improving teaching. She described her changing conception of the role of research in teaching:

I used to have no idea why people would do research and what the significance of research for teachers was. Now I understand that for teachers working in the higher education system, it is part of their work. Just like if you work in a company you need to keep learning and receive training, doing research is also an effective way to enrich teaching. This understanding has a profound influence on me.

The international experience convinced Jane that “teaching and research are complementary in the higher education setting.” Doing theoretical and empirical research can inform teaching, and teaching practice can in turn give rise to theoretical enlightenments. She became willing and happy to invest her time in this useful and interesting theory-practice nexus.

Tony majored in English literature during his study at university. When he graduated and started to teach, he could not find any research interest in this field and therefore, for several years he had no clear research direction. While studying in Singapore, he found that his professors’ research work in applied linguistics was very relevant to his teaching. He suddenly realized that if he could base his research on his teaching and engage in research on language teaching and learning, he would be able to integrate teaching and research, thus achieving a “win-win situation.” For Tony, the Singapore training program not only helped him find his research direction, but also enhanced his knowledge and skills in academic reading and writing as well as in conducting research:

Studying in Singapore had a great impact on me, because in my previous educational experience, I had not received such systematic training in the teaching and research of a subject. I had a total of two semesters of classes there. All the professors who taught us had extensive publishing experience and published many articles in international journals. They incorporated their experience in teaching, research, and writing into their teaching process, which benefited me a lot. Besides, during my study there, I had to read many articles, carry out small research projects, and complete many assignments in English. All of these were new to me and were very helpful.

Working at the university entails taking on the role of an academic who is engaged not only in teaching but also in research. Study abroad allowed the teachers to gain research competence and to see the connections between teaching and research. Current literature about language teachers in study abroad contexts has rarely addressed the particular needs and experiences of university language teachers in terms of research engagement. The findings of this study may contribute to a deeper understanding of the impact of study abroad on language teachers’ academic identity development.

#### From follower to change agent

Before studying abroad, several of the participating teachers did not show much initiative at work. They would follow others and do what they were assigned to do. International professional development experiences allowed the teachers to see the differences between China and the outside world in the field of language education, broadening their horizons and cultivating their desire to bring about change in their professional lives and in their work contexts. Jane was a typical example. As a young female teacher, she used to have no clear career goals and no desire to work hard and excel. Overseas experience transformed her views on life and work, kindling her passion for the profession. She started to treat life and work with more enthusiasm and more positive attitude. When she returned to China, she was full of vigor and passion, actively engaged in various professional development activities, discussed with colleagues about potential innovations in teaching, and tried out research projects to improve her teaching practice. She explained the reasons for her changing agency:

Before I went abroad, I lacked the motivation for professional development. Pursuits such as professional titles did not appeal to me. But when I was abroad, foreign teachers and professors in my office were incredibly hardworking. They often held seminars or international conferences. The intellectual atmosphere was very strong. Colleagues would talk about what they were doing or what books they were reading. This is one of the biggest differences.

Different from Jane, Lucy’s desire to bring about change came from her sense of mission and sense of empowerment as a result of the overseas educational experience:

After going abroad, I felt that I was endowed with a sense of mission. First, I represented China when I was in a foreign country, and I was on Chinese scholarship. I started to ask why I was sponsored to study abroad. At that point, I realized that I needed to do something in return when I came back. Otherwise, I would feel guilty in my heart. Another point is that after all, there have been some changes and improvements in my professional abilities. I wanted to give full play to these abilities, not wasting this experience. So with a combination of these factors, I was very ambitious and very eager to do something when I came back.

Other returnee teachers also expressed the desire to change to varying degrees. Empowered by the overseas experience, they hoped to bring about positive changes in teaching, research, administration, and other aspects of work. Some of them shared their newly acquired knowledge and skills in teaching and research as well as books and resources obtained from abroad with colleagues, involving them in teaching innovation and research projects. Some teachers eagerly tried out new teaching methods to solve problems engrained in college English teaching in China, and some developed new courses using what they had learned and observed abroad. For example, Jessica witnessed the effective practice of team teaching during her stay in the US. When she came back to China, she started to co-teach an online English writing course with a professor she met when she was in America. This course was the first online academic English course offered to undergraduate students at the university and received very positive feedback from students. Jessica also made use of this online course to collect data and carry out teacher research. She shared her innovative teaching practice and research findings by presenting at conferences and publishing articles in top journals in the field, thus inspiring more college English teachers in China to implement similar international collaborative teaching programs.

Li and Edwards (2013) suggested that returnee teachers could serve as “opinion leaders” (p. 402) and contribute to educational innovation by bringing changes to the cognition and teaching practice of teachers around them. This study also found that international experiences sparked the teachers’ desire to effect change in their contexts, but the teachers’ initiatives to effect change were encountered with many barriers and challenges. Indeed, just as past research shows, educational change and innovation are never easy processes (Fullan, 2001). In the Chinese context, it may involve clashes between the old and the new, differences between Western educational philosophies and traditional Chinese values, synergy between individual teachers and the community of practice (Lave and Wenger, 1991; Wenger, 1998) toward a common goal, among a wide range of other influencing factors (e.g., Hu, 2002, 2003, 2005). The complexity involved in this process needs to be further explored.

### Negotiation of professional identity tensions

Although international experiences could bring about positive changes to the teachers’ professional identities, their negotiation of the newly developed professional identities upon return to China was not an easy process. Rather, it was a dynamic, ongoing process of negotiating with the constraints of the personal and professional contexts to which they returned.

One of the most salient identity tensions that the returnee teachers encountered was between the teachers’ efforts to implement the newly acquired Western, communicative, student-centered teaching approach and the constraints of the Chinese teaching reality. Various contextual factors such as students’ levels and learning preferences, the curriculum requirements, and the culture of the institution could affect the teachers’ implementation of new teaching approaches and methods. To negotiate this tension, most of the teachers adopted flexible and creative strategies to achieve a balance between the old and the new approaches. Lucy described the teaching dilemma she faced when she first came back from Britain and the solution she adopted:

When I was studying abroad, I felt that immersion was a good and useful way of language teaching… So after I returned to China, I was very idealistic. I spoke English all the time in class. At that time, my level of English was high and I hoped the students could make it as well. At least they should have the desire to use simple English and create an English-speaking environment to communicate. However, this could not be implemented because of students’ lack of motivation and limited English proficiency. I spoke English with passion, but they hardly understood. Later, I had to adopt bilingual teaching in Chinese and English.

In Lucy’s case, her plan to implement English Medium Instruction (EMI) was challenged by students’ lack of language proficiency and motivation. Realizing this problem, she decided to make compromises and adjustments, switching to bilingual instruction in the end. Lucy also talked about her adjustment in teaching methods:

My teaching methods have changed, undoubtedly different from the way I used to teach before studying abroad, but it does not mean I can make dramatic changes in my teaching. That is not feasible. I can only make adjustments step by step because students have learned English for so many years under the traditional teaching method. Another concern is the differences in students’ English levels. You have to make sure the majority of the students can accept and understand.

Due to consideration of students’ learning styles and familiarity with traditional teaching methods, as well as the enormous individual differences in students’ language proficiency, Lucy could only adopt an eclectic approach, integrating some new elements into the old teaching practice. Other teachers also reported similar strategies of adaptation, adjustment, and balance finding in their teaching. This finding is similar to Li and Edwards (2013) regarding Chinese returnee teachers’ selective uptake of Western teaching methodology to fit the needs of students.

Another major identity tension was between the teachers’ desire to reach out, to collaborate, and to seek support in research and educational innovation and the lack of professional learning community in their work context. For example, Jane’s initial enthusiasm to bring about change right after returning from her international visit soon diminished largely due to lack of encouragement, guidance, and external support. She narrated:

When I first came back to China, I was very enthusiastic. I would talk about this and that with others, about my experiences, and about what I think was particularly good. But I found that there were not many people who were really interested in what I said; instead, they just listened to me politely. My colleagues seemed to be more willing to talk about life rather than academics and teaching. Now I’m back on the old path. I chat with my colleagues on weekends, but I do not even know what I’m talking about!

Jane tried out some research projects when she came back to China, but unfortunately these research efforts did not materialize and sustain. When reflecting on the reasons, she mentioned that she missed the academic atmosphere of the Dutch university she visited, which was very supportive and inspiring:

After I came back, I did some simple, small-scale pilot research in teaching. I also did a few questionnaire surveys, but none of them took shape. The reason for my lack of concentration and persistence in research is that I sometimes encountered bottlenecks and did not know what to do next. Apart from personal reasons, my slow progress also had to do with the environment. Personally, I prefer to have discussions to generate more ideas, and I like to hear others’ voices. In this regard, I really enjoy the academic atmosphere of my overseas program.

Jane’s story echoed the experiences of many returnee teachers. Indeed, international experiences may afford the life and work of returnee teachers new meanings, transforming their personal and professional identities. In this process, they became somehow disconnected from the old environment and needed to join new professional learning community, which may not be available in their work context. To fill this gap, some of the teachers took the initiative to network with like-minded colleagues, some kept contact and collaborated with professors they met when abroad, and some joined not only local, but also transnational and virtual professional learning communities to sustain their professional development.

The third identity tension had to do with the teachers’ desire to carry out academic research and make educational change constrained by personal factors such as lack of agency, limited time and energy, and inadequate knowledge and skills. Tony explained why he was slow in doing research upon return to China:

There may be many reasons for the slow progress of my research. One is the teaching load. I teach many classes, so I am relatively busy. There are also family obligations such as picking up and taking children to school. The main reason should be about myself. I did not make effective use of my time and did not arrange my time very well. Therefore, I always feel I do not have enough time ... I’m also kind of lazy. In addition, in the process of doing research, I always feel inadequate. I feel this part is not mature and that part is not ideal. So my research work has not progressed very smoothly.

Tony was eager to carry out innovative research after witnessing how his professors in Singapore were passionate about and fruitful in research. However, when he returned to China, he found that doing research was a very slow and challenging process. Apart from environmental constraints such as lack of guidance from experts and different research priorities between China and the international community, his research work was slowed down by his teaching duties and family roles as well as his inadequate knowledge and skills to carry out research independently. Fortunately, Tony was very persistent and focused in his research work. He sought out various opportunities to improve his research capacity and kept in close contact with professors in Singapore. Later on, he successfully published several articles in collaboration with his professors.

It should be noted that the overseas experience might have the function of awareness raising and knowledge enrichment for the language teachers, but it may not be sufficient in helping the teachers become competent, independent researchers and educational innovators. Upon return, the teachers needed to exert strong agency to negotiate personal, interpersonal, and contextual constraints and to engage in continued professional learning in order to sustain professional development and growth. This finding about the strong link between identity and agency is consistent with previous research on language teacher identity (Tao and Gao, 2017; Hiver and Whitehead, 2018). To sustain the returnee teachers’ agency for continued professional development, ongoing, systematic support is highly necessary and desirable. Relevant discussion about how to support the long-term development of language teachers after the completion of study abroad programs is scarce in the current research on language teacher study abroad, and needs to be strengthened to maximize the effect of study abroad for the professional development of language teachers.

## Conclusion

This study has found that international experiences had a positive impact on Chinese college English teachers’ professional identity construction by empowering them to develop multiple professional identities as language teaching professionals, university academics, and change agents. As Wenger (1998) suggested, learning is paramount for identity construction because “it transforms who we are and what we can do” (p. 215). The findings of this study have indicated that international experiences are an important tool for teacher learning and teacher development, but it cannot be assumed that positive changes will naturally occur after the teachers’ stay in an overseas environment, because the process of teacher learning and identity development is highly complicated. For the returnee teachers, it is a process of negotiating their newly developed professional identities with the realities of the context, which is mediated by the interplay of various personal (e.g., agency, time, and energy), interpersonal (e.g., professional learning community), and environmental factors (e.g., institutional realities). The current study contributes to the limited body of research on the professional identity development of internationally trained language teachers, especially in-service teachers working in university settings, adding to our understanding of the role of international experiences in the professional development of second language teachers in similar contexts.

The study has several implications. For international professional development programs, in addition to considering the fit between the program orientation and the local contexts of the participating teachers, it might be important to consider the “after program” effect and to explore feasible ways to build into its curriculum design the support mechanisms for the teachers’ long-term professional development so that the teachers may be equipped with the necessary tools and strategies to better cope with potential barriers and challenges upon return to home country. Activities such as discussions and self-reflections on identity issues and study abroad impacts can be offered to raise teachers’ awareness of how identity may be shaped by changing contexts over time and what strategies can be adopted to connect the past, the present, and the future to facilitate their professional identity development. For the home institution, a nurturing environment with ongoing support is necessary to maintain the returnee teachers’ agency for continued professional development and to facilitate the teachers’ successful negotiation of new professional learning with the realities of the work context. It should be noted that returnee teachers may not be ready to take on independent research work or engage in innovative teaching programs simply because they have received training abroad. They need strong and sustained support from the outside, which may take the forms of funding, resources, further professional development opportunities, and understandings of the particular experiences and needs of overseas returnee teachers.

Finally, as an exploratory case study, the findings reported here are limited by the small sample size and the predominant data source of teachers’ self-reports. Future research can draw upon larger sample size or mixed-methods design, and triangulate the perspectives of other stakeholders such as teacher educators, program administrators, and students, in order to expand our understanding of the complex process of professional identity development of language teachers as a result of study abroad.

## Data availability statement

The raw data supporting the conclusions of this article will be made available by the authors, without undue reservation.

## Ethics statement

The studies involving human participants were reviewed and approved by Jilin Educational Research Office. The patients/participants provided their written informed consent to participate in this study.

## Author contributions

JW drafted the literature review and methods sections and assisted in data analysis. FZ collected and analyzed the data, drafted the other sections of the manuscript, and finalized the manuscript. All authors contributed to the article and approved the submitted version.

## Funding

This work was supported by Jilin University Faculty Teaching and Development Project, Jilin Province 12th Five-Year Educational Plan Project, and International Innovative Team Project (2019GJTD02).

## Conflict of interest

The authors declare that the research was conducted in the absence of any commercial or financial relationships that could be construed as a potential conflict of interest.

## Publisher’s note

All claims expressed in this article are solely those of the authors and do not necessarily represent those of their affiliated organizations, or those of the publisher, the editors and the reviewers. Any product that may be evaluated in this article, or claim that may be made by its manufacturer, is not guaranteed or endorsed by the publisher.
